# Intelligent Bar Chart Plagiarism Detection in Documents

**DOI:** 10.1155/2014/612787

**Published:** 2014-09-17

**Authors:** Mohammed Mumtaz Al-Dabbagh, Naomie Salim, Amjad Rehman, Mohammed Hazim Alkawaz, Tanzila Saba, Mznah Al-Rodhaan, Abdullah Al-Dhelaan

**Affiliations:** ^1^Faculty of Computing, Universiti Teknologi Malaysia, 81310 Skudai, Johor, Malaysia; ^2^Faculty of Computer Sciences and Mathematics, University of Mosul, Mosul, Iraq; ^3^MIS Department, CBA, Salman Bin Abdulaziz University, Alkharj, Saudi Arabia; ^4^College of Computer and Information Sciences (CCIS), Prince Sultan University, Riyadh, Saudi Arabia; ^5^Computer Science Department, College of Computer & Information Sciences, King Saud University, Riyadh, Saudi Arabia

## Abstract

This paper presents a novel features mining approach from documents that could not be mined via optical character recognition (OCR). By identifying the intimate relationship between the text and graphical components, the proposed technique pulls out the Start, End, and Exact values for each bar. Furthermore, the word 2-gram and Euclidean distance methods are used to accurately detect and determine plagiarism in bar charts.

## 1. Introduction

Detection, determination, and rectification of plagiarism are outstanding quests in every sphere of documentation and copyright. Lately, the significant advancement in information technology represented by digital libraries and World Wide Web is regarded as one of the main reasons for exponential growth in plagiarism appearance. It has become effortless for the plagiarist to utilize or copy the work of others without acknowledging or citing them due to the easy availability of most resources in digital format. Thus, plagiarism is regarded as one of the electronic crimes and intellectual thefts from others documents [1–3]. In academia, plagiarism posed a severe educational challenge which is acutely faced by research institutions, universities, and even schools. Several efforts are dedicated to detecting different types of plagiarism via programming code and text. Plagiarism detection began in the 1970s, where the identification of rate of plagiarism in programming code written by some computer languages such as C and Pascal was introduced [4]. Digital documents being the major carriers of information require extreme authentication in terms of their origins and trustfulness. The quest for achieving an accurate and efficient image forgery detection method in digital documentation is never ending. Developing a robust plagiarism detector by overcoming the limitations associated with human intervention is the key issue [5].

Recently, several researchers developed the algorithmic approach using computer codes to detect plagiarism in the homework of students [6]. Based on levels of plagiarism patterns some studies introduced plagiarism detection methods which are implemented in the algorithms and program codes [7]. Generally, the computerized or statistical approaches are exploited to detect plagiarism in natural language since the 1990s. The techniques used for natural language are based on various factors such as grammar, semantic, and grammar-semantics hybridizations [1, 4]. However, the grammar-based method is one of the restrictive ones to detect plagiarism. This type of method analyzes the sentences based on grammatical structure, which can be efficiently used to detect the Exact Copy of text. While semantic-based method utilizes vector space model to calculate the similarities among the texts. Undoubtedly, the grammar-semantics hybrid approach overcomes all disadvantages of the other methods. This is considered as one of the most versatile techniques to detect text plagiarism [1, 8, 9].

A new taxonomy is introduced to explain the concepts for various types and patterns of text plagiarism [4]. Plagiarism is divided into two main parts including literal and intelligent one. Each part consists of several subparts which cover all possibilities of text plagiarism. Generally, the representations of quantitative information are formulated via infographic form by using figures, charts, and tables. The information that is displayed in charts, figures, and tables includes results of experiments, framework, and statistical facts. These data and information in homogenous form can be formulated by using various shapes such as pie chart, bar chart, and 2D and 3D plots [10–12].

We report a new type of plagiarism detection method by highlighting the types of information that can be stolen from others work without referencing. Firstly, different types of forged information are organized into taxonomy of chart, figure, and table to highlight varieties of plagiarism patterns such as Exact and Modified Copy. Secondly, plagiarism detection in bar chart image is performed depending on ten features in images. Some of the features are extracted by OCR tool while others are acquired from the relationship of text and graphic components [13, 14]. Finally, the proposed technique is used to extract the features of bar chart images which cannot be extracted by OCR to detect plagiarism. The paper is organized as follows: Section 2 describes various existing techniques for extracting data from bar chart images [15]. The taxonomy of chart, figure, and table related to plagiarism is presented in Section 3.  Section 4 discusses the methodology and Section 5 includes the experimental results of bar chart plagiarism detection. The discussions are elucidated in Section 6.  Section 7 concludes the paper.

## 2. Related Work

Categorizations of bar chart images refer to their labeling into one of the predefined geometrical or nongeometrical classes. Though the classification is apparently manageable, it is proven to be an extremely difficult problem in computer programming. Hence, there is an intense attention in developing automatic tools to categorize, describe, or retrieve images based on their contents.

Consequently, researchers attempted to extract the features and data from chart images. For automatic images categorization and description computational model is successfully introduced [16]. The analysis of local and global image characteristics by using text and image features is used in the model. The model is capable of differentiating geometrical and ordinary images. The computational model is comprised of classifier stage which is trained by the associated text features using advanced concepts and similarity matching stage.

Classification methods based on multiple-instance learning for chart images are also developed [10]. A re-revision system consisting of three concatenated major stages such as classification, extraction, and redesigned chart images is employed [17]. In the extraction stage, two types of charts (pie and bar) are focused on. Some techniques are presented in extracting data and graphical marks from chart images. Truly, the understanding and recognition of chart images require the preprocessing and extraction of data and information. Primarily, two types of available methods that deal with chart images are either to consider electronic chart directly [18–20] or to obtain them after converting into raster images [21–24]. Mishchenko and Vassilieva [25] introduced a model-based method for the classification of chart images which involved two main stages: firstly, predicting the location and the size of chart depending on the color distribution of chart image and secondly the extraction and matching of chart image edges to achieve the best match between query and database images.

The techniques for features extraction of image depend on the type of images such as chart or medical representation. Some techniques are applicable on two-dimensional plot of chart images while others work well for bar chart images. Hough transform technique is introduced as an approach to extract the features of bar chart images [23]. Some investigations are based on the edges of bars to extract the features [24, 26]. The learning-based method is established to recognize the chart images [22]. The features of bar chart images can be extracted by describing the height and width of each bar, which is applied on statistical images to determine the similarities [27]. Meanwhile, other techniques focused on geometric features rather than data and information of scientific bar chart images [28].

Currently, several techniques are developed to extract the features from medical images. The texture is one of the visual contents of a medical image used in content-based image retrieval (CBIR) to represent the image effectively for searching and recovering similar areas [29]. Gray-level statistical matrix technique is applied to extract the texture information for the content-based retrieval of mammograms from the MIAS database [30]. 3D texture features technique based on the cooccurrence matrixes of the gray-level, gradient, and curvature information regarding the nodule volume data for classifying the malignancy from benign is introduced [31, 32].

## 3. Plagiarism Taxonomy and Patterns

Three types of graphic plagiarism such as figure, chart, and table are important to emphasize. Each type highlights different levels of plagiarism. The patterns and types of plagiarism for figures, charts, and tables are presented as taxonomy. Some kinds of text plagiarism are also evaluated [33]. The methods for detecting passages of text plagiarism for documents without appropriate citations are also suggested. Taxonomy is further extended to cover other types of plagiarism [4]. The taxonomy presented in various studies majorly demonstrates literal and intelligent plagiarism, where each kind includes many patterns of plagiarism. However, we are interested in detecting plagiarism of charts, as well as their taxonomy, figures, and tables. Alternatively, charts (pie, bar, and line) can be considered as one of the methods for representing the data and information of experimental results or comparing among techniques which are copied from other references without citation. Therefore, plagiarism of charts can be formulated in several forms to manifest the same information in various shapes. Taxonomy of chart plagiarism demonstrates many patterns and models which may be used to plagiarize the data of chart image.

Plagiarism patterns of chart, figure, and table are divided into Exact Copy and Modified Copy prototypes. The Exact Copy patterns of plagiarism are defined as the direct quote of data from other works without referencing, where copy and paste of the whole or part of the information image is performed. Simplicity is one of the important attributes of this type of plagiarism. Besides, this type of plagiarism does not require much time to hide the academic crime. The other type of graphic plagiarism is the Modified Copy for information of chart, figure, and table. This is more intelligently performed than the previous one because the same data can be formulated in many ways to exhibit the work in a different style than the original one. The goal of these intelligent means is that the plagiarist attempts to deceive the readers by doing some changes, such as translation from other languages or generating another shape for the same data.

**Figure 1:**
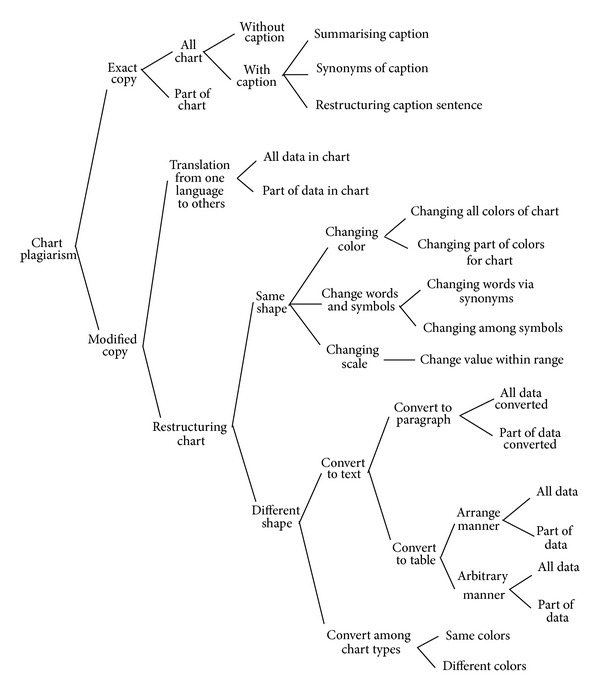
Taxonomy of chart plagiarism.

**Figure 2:**
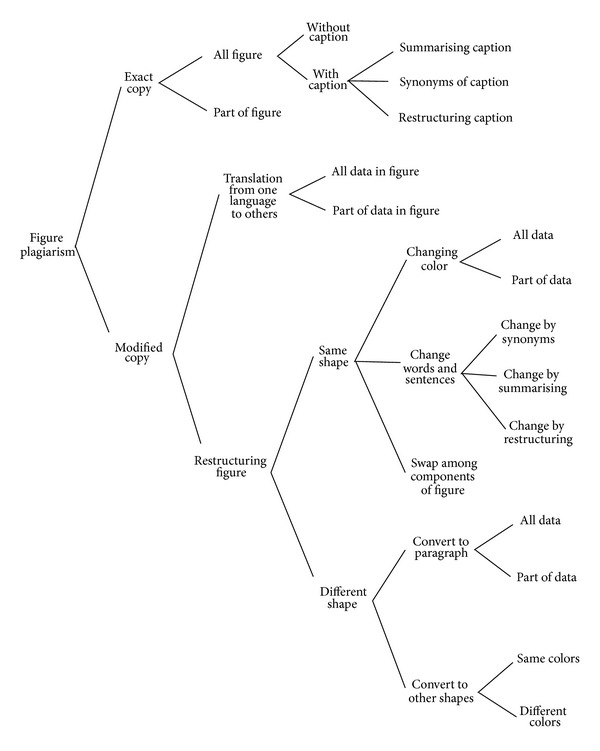
Taxonomy of figure plagiarism.

**Figure 3:**
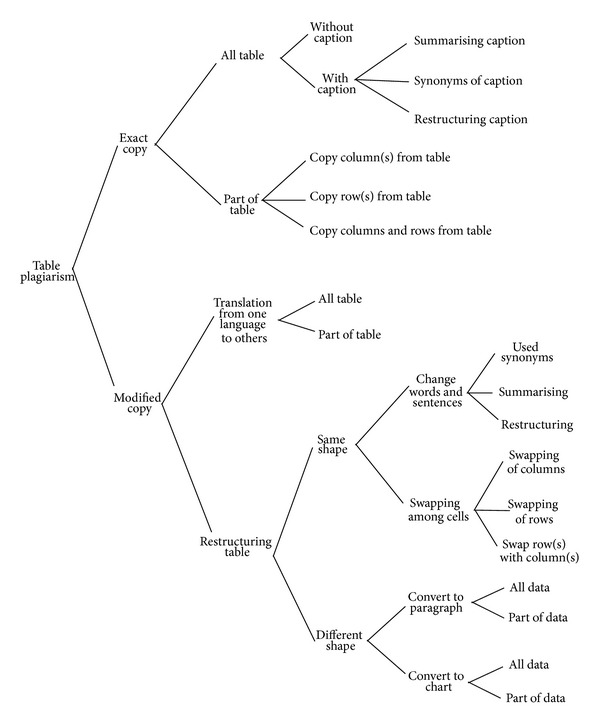
Taxonomy of table plagiarism.

The Modified Copy plagiarisms are primarily divided into translation and restructuring. In this research, new types of copying are organized by taxonomy which explains various patterns of graphic plagiarism. Furthermore, the primary focus of the bar chart image is to detect the proportion of plagarism. Figures 1, 2, and 3 depict the taxonomy of chart, figure, and table plagiarism, respectively.

## 4. Methodology

**Figure 4:**
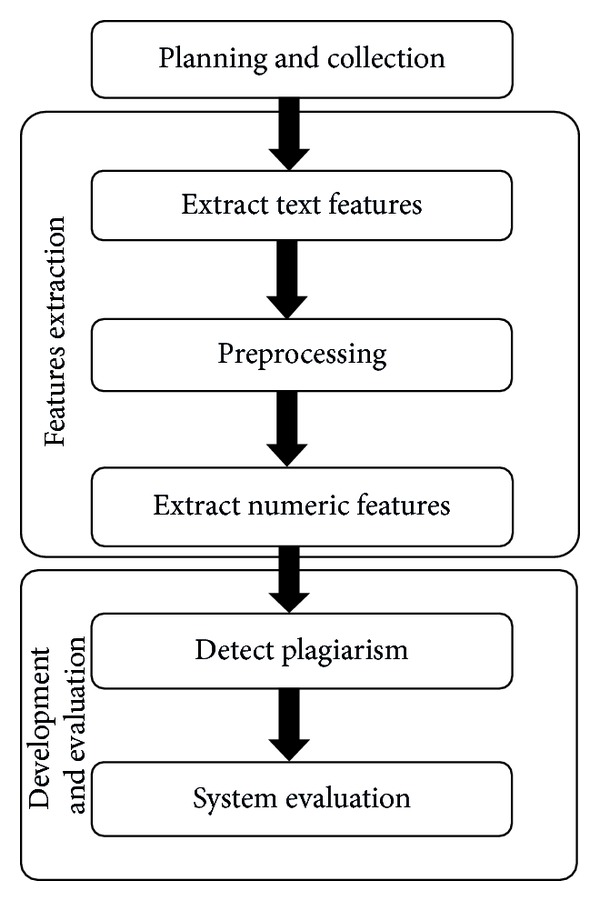
Flowchart of methodology.

**Figure 5:**
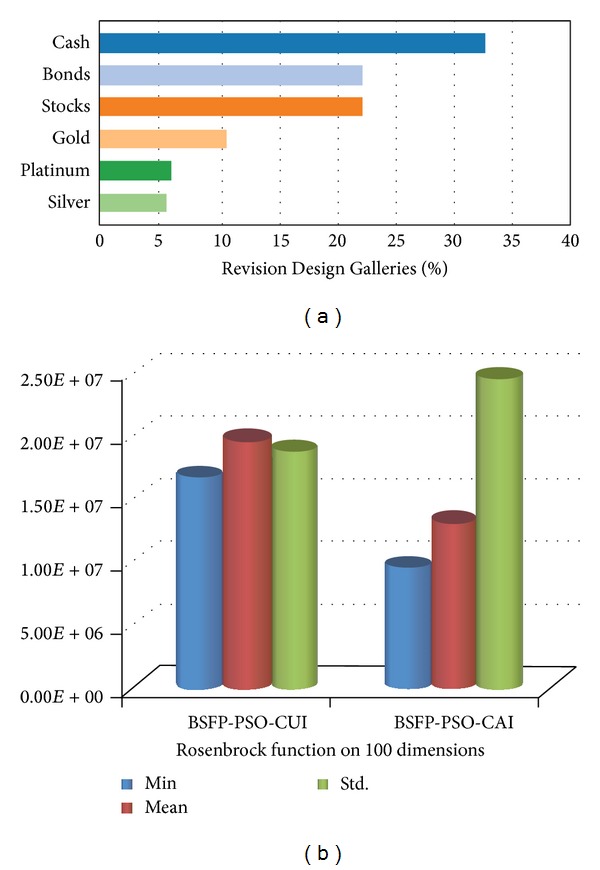
Samples of data set [17, 36].

The methodology of bar chart plagiarism detection as shown in Figure 4 consists of three main stages, namely, planning and collection, feature extraction, and development with system evaluation. In the planning and collection stage, various patterns of graphical plagiarism via taxonomy of chart, figures, and table are presented (Figures 1, 2, and 3). The taxonomy of chart plagiarism explains different formulations which plagiarize the data of bar chart images. Therefore, varieties of bar chart images are collected and data sets are considered. The gatherings of the data sets consist of 100 bar chart images for storing in databases and twenty images for query including all possibilities of plagiarism for bar chart images. These data sets are collected from different resources such as thesis, which represented various types of bar chart images in 2D and 3D. Besides, vertical and horizontal bar chart images as shown in Figure 5 are taken into account.

In the feature extraction stage, the features of bar chart images are used to detect plagiarism. Various types of bar chart images are analyzed to detect the features of image. The bar chart images inferred to acquire maximum of ten features representing the information and the data of image. These features are common in different types of bar chart images, for instance, in 2D and 3D images. However, the number of uses for these features may different from each other.

The features extraction is an essential process to get the data from images which can be utilized to detect the rate of plagiarized data. Therefore, these ten features are categorized into low-level and high-level features. The low-level features refer to the text features of bar chart. The text features are the text which can be used in image to represent the information and data such as caption of image, label of each bar, label of coordinates, and values on coordinates. Generally, they are extracted from bar chart images using OCR tool. Conversely, the high-level features referring to numeric features cannot be extracted using OCR tool. The extraction of numeric features requires a relationship between the text and graphic components. The numeric features include values of bars in image. Each bar in an image has three numeric features which can be extracted by the proposed technique depending on Start, End, and Exact values. The Start and End values represent the first and last values while the Exact one corresponds to the real value of the bar. For instance, the text features for image in Figure 5 are* “Figure 10: Revision Design Galleries,” “Silver, Platinum,…, Cash,”* and* “0%, 5%,…, 40%”* which represent caption of image and label of coordinates* X* and* Y*, respectively. Meanwhile, the numeric features represent the value of each bar, which can be detected by Start, End, and Exact features. For example, the Start, End, and Exact features of bar Silver are 5%, 10%, and 6%, respectively.

These features are used to detect the proportion of plagiarism for bar chart image. The extraction of Start, End, and Exact values necessitates preprocessing of bar chart image to the adjacent coordinates of the image. Image scanning is then performed to detect the length of each bar in order to find the numeric features for each bar. Storing of the features in databases depends on the type of features whether numeric or text. The features which are extracted by the proposed technique are represented as vectors, while the text features that are extracted by OCR are characterized as string.

The detection methods for text plagiarism are mainly categorized based on character, semantics, structure, citation, cluster, cross language, and syntax. Comparatively, the smaller number of textual components than normal paragraph text allows us to use character-based methods to detect plagiarism of bar chart images. The character-based methods depend on character matching approaches to exactly or partially detect the identical string for features of bar chart images. Various algorithms of plagiarism are adopted in the text as character *n*-gram to identify the similarity between two strings based on the number of identical characters of features. Some researchers use 8-gram and 5-gram techniques [34, 35] for matching strings to detect plagiarism. We used 2-gram technique to detect plagiarism of bar chart images. This technique is used to represent the text features of bar chart images. Different similarity measures can be used to obtain the similarity for numeric features such as Euclidean distance, Jaccard, or cosine coefficient. The Euclidean distance is calculated by the following:

(1) $$\begin{array}{c} \text{Ec}\left(x,y\right)=\sqrt{\sum_{i}{\left|x_{i}-y_{i}\right|}^{2}}. \end{array}$$

Once the detection and storing of the proportion of plagiarism are completed then the performance of the system is evaluated. The performance is evaluated by overlapping of features using the relation of Precision and Recall given by the following:

(2) $$\begin{array}{c} \text{Recall}=\;\frac{\text{Relavent}\;\text{Documents}\;\text{Retreived}}{\text{All}\;\text{Relavent}\;\text{Documents}},\\ \text{Precision}=\;\frac{\text{Relavent}\;\text{Documents}\;\text{Retreived}}{\text{All}\;\text{Documents}\;\text{Retrived}}. \end{array}$$

## 5. Experimental Results

The bar chart plagiarism detections are carried out in four main stages such as submission of query images, feature extraction of bar chart image, plagiarism detection, and highlighting results. The first stage is to submit various types of query images covering different kinds of possible plagiarism to detect and judge plagiarism of bar chart images, while the features of query are extracted in the second stage. The third stage includes detection of plagiarism by using word 2-gram and Euclidean distance techniques. Finally, the features of query bar chart image that are plagiarized from others are highlighted and the proportion of the similarity is displayed.

**Figure 6:**
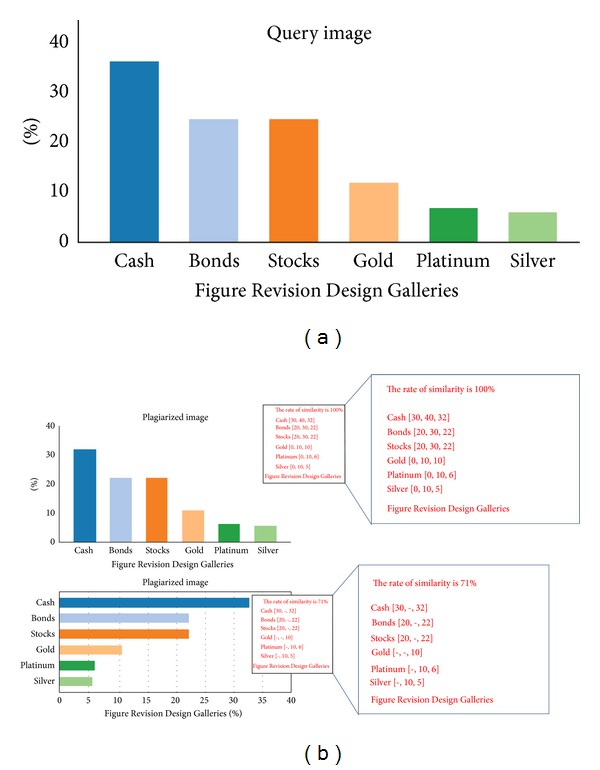
Plagiarism detection for one of Exact Copy patterns as plagiarism of the whole data of image.

The bar chart plagiarism is further divided into* Exact Copy* and* Modified Copy* as explained in taxonomy.  Figure 6(a) shows the query image while Figure 6(b) depicts the plagiarized images detected by the system. The first plagiarized image is similar to the whole data in the image while the second plagiarized image contains the same data that was plagiarized but presented as a horizontal bar chart image. The system extracts the features of query image and detects the proportion of plagiarism depending on Start, End, and Exact values for each bar as well as the label of each bar. The system highlights the data and information that are plagiarized and provides the proportion of plagiarism.

**Figure 7:**
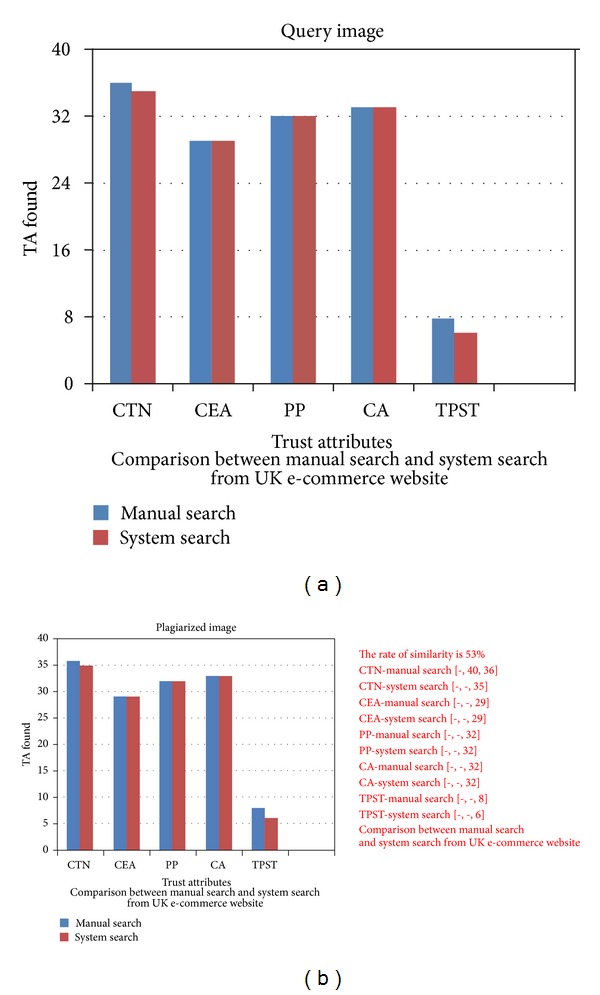
Plagiarism detection for one of the Modified Copy patterns which is the stealing by changing scales of image.

One of the patterns of plagiarism derived from* Modified Copy* is the stealing by changing scales. Each bar is modified by plagiarists by changing Start and End values to be different from the original image.  Figure 7 illustrates the significant role played by the Exact values to detect this type of plagiarism.

**Figure 8:**
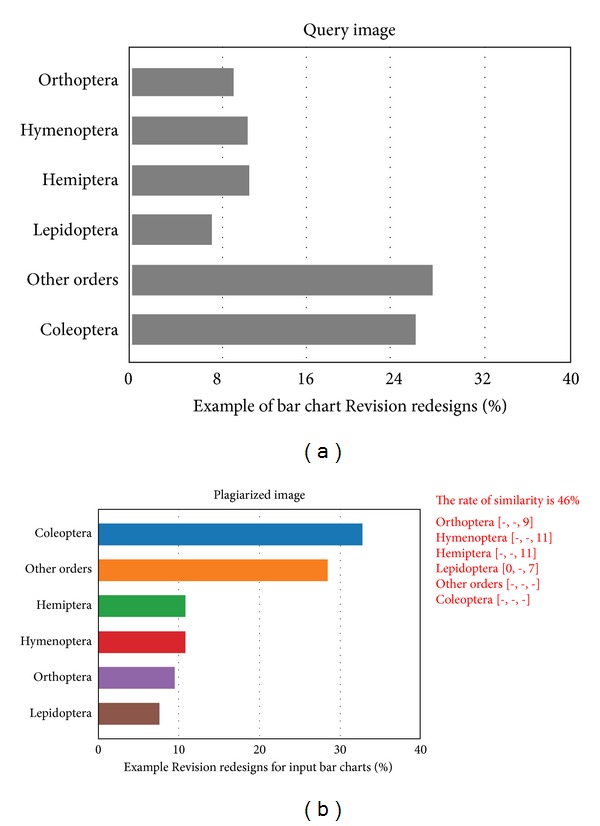
Plagiarism detection for integration among possible bar chart plagiarism.

The plagiarists may use integration among patterns of possible bar chart plagiarism to present a more complex image which has the same data quoting from other works.  Figure 8 displays the query image which is modified by changing colours and scales of bars as well as changing their location via swapping. The proposed system is capable of detecting this type of plagiarism and identifies the proportion of similarity.

**Figure 9:**
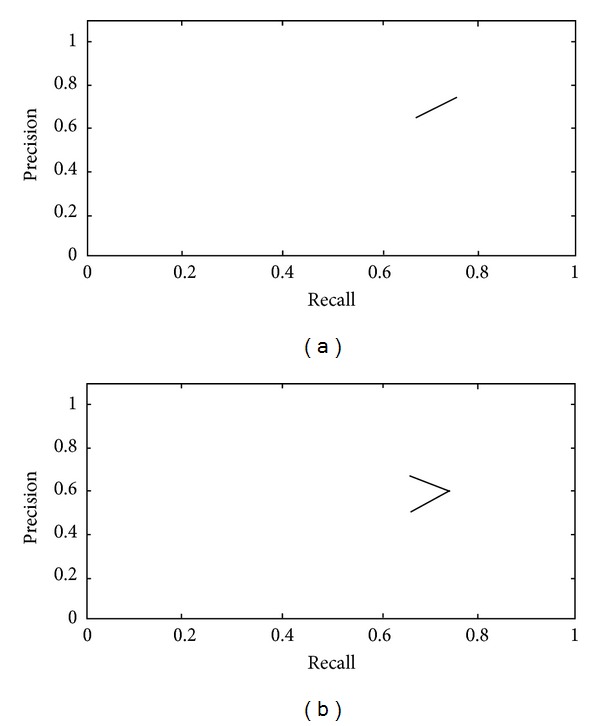
The evaluation of performance (a) for* Exact Copy* patterns and (b) for* Modified Copy* patterns.

Figure 9 illustrates the performance of the system for plagiarism detection of* Exact Copy* and* Modified Copy* patterns, respectively.

## 6. Discussion

The state-of-the-art graphical plagiarism techniques and patterns are presented. The graphical plagiarism is considered one of the electronic crimes and thefts and the concepts of such stealing are newly viewed. Various important information and data can be represented as graphical forms such as results or frameworks for academic and business aspects. However, many systems of text plagiarism methods such as Turnitin are incapable of detecting plagiarism of images. In spite of the different styles of bar chart images, the extraction of features of image plays an important role in detecting plagiarism. Our proposed technique which is used to extract the numeric features played an essential role for bar chart plagiarism detection. The patterns of* Exact Copy* of bar chart plagiarism detection including direct copy of all data or part of data are underscored. Plagiarism which is carried out by modifying caption of images via restructuring or summarizing for label sentences is emphasized. Alternatively, the patterns of* Modified Copy* which are regarded as more complicated than* Exact Copy* patterns are also analyzed. The difficulty of these patterns is the changing on image which appears as the same data and information in different forms. The restructuring of information for image within the same shape is also covered. The edition of bar chart images including the change of image bar colors or changing the bar locations either by swapping or via generating horizontal bars from vertical bars and vice versa is discussed in detail. Besides, more professional modification such as changing of scales on coordinates which is completely different from original one can be detected by the proposed method. In this case, the* Start* and* End* features of bars are completely different. Consequently, the* Exact* features as well as other attributes play significant role in detecting plagiarism in bar chart image.

## 7. Conclusion

We demonstrate the precise recognition of different plagiarized patterns in business documents using an intelligent bar chart detection system. The types and patterns of plagiarism are presented via taxonomy of figure, chart, and table. Various kinds of possible plagiarism are highlighted using taxonomy. Plagiarism of bar chart image as type of chart is detected by the newly proposed technique. It is established that the present technique is capable of extracting the features from a bar chart image which cannot be pulled out using OCR tool. Our technique first recognizes the connection between the text and graphical components to extract the Start, End, and Exact value for each bar. Using word 2-gram and Euclidean distance methods the accurate detection of plagiarism is performed. The detection of plagiarism is based on ten striking features. The system is capable of detecting different levels of plagiarism not only copy and paste of bar chart image but also modification on images such as changing color or scales. The present system efficiently and accurately distinguishes other possible alteration administered on these images such as swapping among bars location and even changes on caption via summarizing and restructuring. The proposed technique may be useful for intelligent plagiarism detection in business and academic documents.
